# Kidney injury associated with COVID-19 infection and vaccine: A narrative review

**DOI:** 10.3389/fmed.2022.956158

**Published:** 2022-12-05

**Authors:** Iago Carvalho Rezende Pacheco, Denise Maria do Nascimento Costa, Deborah Serra Sousa, Natalino Salgado Filho, Gyl Eanes Barros Silva, Precil Diego Miranda de Menezes Neves

**Affiliations:** ^1^Division of Nephrology, University Hospital of the Federal University of Maranhão, São Luís, Brazil; ^2^Division of Nephrology, Hospital das Clínicas of the Federal University of Pernambuco, Recife, Brazil; ^3^Ribeirão Preto Medical School, University of São Paulo, São Paulo, Brazil; ^4^Division of Nephrology, University of São Paulo, São Paulo, Brazil; ^5^Nephrology and Dialysis Center, Oswaldo Cruz German Hospítal, São Paulo, Brazil

**Keywords:** COVID-19, SARS-CoV-2, acute kidney injury, COVAN, kidney biopsy, vaccination, kidney morphology

## Abstract

The respiratory tract is the main infection site for severe acute respiratory syndrome coronavirus 2 (SARS-CoV-2), resulting in many admissions to intensive care centers in several countries. However, in addition to lung involvement, kidney injury caused by the novel coronavirus has proven to be a significant factor related to high morbidity and mortality, alarming experts worldwide. The number of deaths has drastically reduced with the advent of large-scale immunization, highlighting the importance of vaccination as the best way to combat the pandemic. Despite the undeniable efficacy of the vaccine, the renal side effects associated with its use deserve to be highlighted, especially the emergence or reactivation of glomerulopathies mentioned in some case reports. This study aimed to identify the main renal morphological findings correlated with COVID-19 infection and its vaccination, seeking to understand the pathophysiological mechanisms, main clinical features, and outcomes.

## Introduction

Since the description of the first report of severe acute respiratory syndrome coronavirus 2 (SARS-CoV-2) in December 2019, the upper respiratory tract has been well established to be the main infection site; however, much evidence has demonstrated that other organs, including the heart, liver, and kidneys in addition to the respiratory tract, can be severely affected (1). Acute kidney injury (AKI) caused by the new coronavirus is associated with the severe clinical status of patients and, consequently, a worse prognosis (2). Although the mechanism of injury is not completely understood, it is currently known to go beyond acute tubular necrosis secondary to hemodynamic instability in critically ill patients (2).

The binding of the viral S protein to the angiotensin-converting enzyme 2 (ACE2) receptor on the surface of host cells triggers SARS-CoV-2 infection. These receptors are in large quantities in type II pneumocytes in the lungs, heart, and kidneys. The virus incorporation into the cell occurs when proteins present on the surface of the virus, called “spikes,” bind to ACE2 and are endocytosed by activating transmembrane serine protease type 2 (TMPRSS 2), which starts the intracellular viral replication (3).

Studies from autopsies have shown that the kidneys are a special target organ of the virus, even in patients without a history of kidney disease. This is probably due to the high expression of proteins, such as ACE2, TMPRSS 2, and cathepsin L that enable viral infection (4). Detection of viral fragments in urine by polymerase chain reaction (PCR) was present in 21–50% of infected patients in the second or third week after infection, suggesting the possibility of renal tropism by the virus (5).

The infection course, morbidity, and mortality have changed favorably since the advent of vaccines against the new coronavirus in the last year, drastically reducing the number of deaths. However, with the spread of vaccination, adverse effects of vaccines, including kidney injury, have generated concerns globally (6). Since the beginning of large-scale immunization, the publication of case series of renal diseases with the emergence of new glomerulopathies or reactivation of previous glomerulopathies has increased; however, the related mechanisms, risk factors, and long-term consequences are not yet well established (7).

### Search strategy

This review aimed to highlight and describe the main morphological and pathophysiological aspects of kidney injury described in the most recent publications related to SARS-CoV-2 infection and after the administration of vaccines against the new coronavirus. This narrative review was based on a comprehensive literature search on PUBMED/MEDLINE, PUBCOVID19, and GOOGLE SCHOLAR databases. The keywords related to “Glomerular,” “Glomerulopathy,” “Kidney,” “Tubular,” “Proteinuria,” and “COVID-19,” “SARS-CoV-2,” and “SARS-CoV vaccine” were used with Boolean combinations.

## Morphological kidney findings associated with COVID-19 infection

Acute kidney injury in patients infected with COVID-19 has proven to be one of the main risk factors for worse prognosis in intensive care units (ICU). Despite studies showing a variation of 0.5–56% in AKI incidence in patients infected with SARS-CoV-2, this high frequency and association with unfavorable outcomes have been consistently reported in studies (8, 9).

Renal involvement ranges from mild proteinuria (44%) and microscopic hematuria (27%) to AKI requiring renal replacement therapy (RRT) (10). Approximately 20% of patients admitted to the ICU required RRT within 15 days after the onset of the disease, but the mechanisms leading to AKI are still not well established (11). Numerous studies have proposed multifactorial etiologies for renal involvement, highlighting hemodynamic instability caused by severe viral infections as the main factor. Other mechanisms, such as the renin-angiotensin-aldosterone system imbalance, dysregulation of the complement system cascade, pro-coagulant status, and release of pro-inflammatory mediators (“cytokine storm”), were also associated (9).

However, some studies have also proposed other etiologies of kidney injury, highlighting the direct viral action on the tubular epithelium and podocyte cells through the ACE2 receptor, causing mitochondrial dysfunction, acute tubular necrosis, and glomerulopathy (11).

### Glomerular injury

**FIGURE 1:**
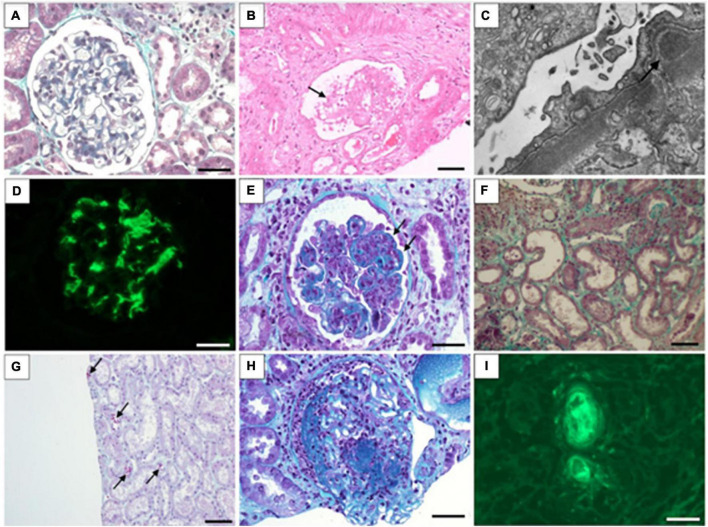
Morphological findings associated with COVID-19 infection in kidney biopsy. **(A)** Normal glomerulus in minimal change disease (Masson’s trichrome stain); **(B)** glomerular collapse and podocytes hyperplasia (arrow) in collapsing glomerulopathy (hematoxylin and eosin stain); **(C)** Subepithelial deposits in membranous nephropathy (electron microscopy); **(D)** Intense mesangial IgA deposits in IgA nephropathy (immunofluorescence); **(E)** Endocapilar proliferation and wire-loop hyaline deposits in class IV lupus nephritis (Masson’s trichrome stain); **(F)** Flattened tubular epithelial cells in dilated tubules in in acute tubular injury (Masson’s trichrome stain); **(G)** Ectatic tubules and ducts contain red-orange granular casts caused by rhabdomyolysis (Masson’s trichrome stain); **(H)** Glomerular necrosis and crescent formation in cases of immune complex-mediated crescentic glomerulonephritis (Masson’s trichrome stain); **(I)** Fluorescence shows intravascular thrombi in thrombotic microangiopathy (detected with antibody to fibrinogen). **(A,D,H,I):** Barr = 20 μm; **(B,G,F):** Barr = 50 μm.

Regardless of the presence of AKI, glomerular injury is an important cause of renal injury during COVID-19 infection (12). Many studies have been published showing that the most common glomerular injury type is podocytopathies, with collapsing glomerulopathy (CG) being the main cause of nephrotic syndrome associated with virus infection (13). However, it became clear after evaluating a series of biopsied cases in which glomerular involvement comprised a wide spectrum of lesions (Figure 1), including membranous glomerulopathy, minimal lesion disease, immunoglobulin A (IgA) nephropathy, and non-collapsing focal and segmental glomerulopathy (14).

In this sense, a renal biopsy is an essential tool in the context of SARS-CoV-2 infection since it identifies the histological diagnosis of glomerular disease and well as highlights the wide variety of other possible histopathological diagnoses (15).

### COVID-associated nephropathy

Collapsing glomerulopathy was first described in the context of human immunodeficiency virus (HIV) infection and was later recognized as “HIV-associated nephropathy” (HIVAN). Subsequent studies have shown that the presence of the high-risk genotype for APOL-1 (HRG-APOL1) in African American individuals significantly increased the risk of developing HIVAN by 30–90% (16). It is currently known that other types of viral infections, including parvovirus B19, cytomegalovirus, and Epstein–Barr virus, also increase the risk of developing CG. Some studies have also pointed out that the common factor in CG cases may be the activation of interferon, given the presence of endothelial tubuloreticular inclusions characterized as “interferon footprints” (16).

In the context of COVID-19, many patients are diagnosed with COVID-associated nephropathy (COVAN), particularly those with HRG-APOL1. The presence of this genotype increases the risk of interferon-mediated podocyte injury in the presence of viral infections (8). In a study of 23 patients who presented with CG after SARS-CoV-2 infection, 91% of the patients were black. Furthermore, of the 17 patients who underwent genotyping, 16 (94%) presented HRG-APOL1. In the follow-up, seven patients with COVAN who required RRT managed to stay off dialysis; however, the prognosis regarding proteinuria and chronic kidney disease remained reserved (17).

In a study with six patients of African–American descent infected with the new coronavirus who had AKI and nephrotic proteinuria, the most prevalent diagnosis found in kidney biopsies was CG with extensive effacement of podocyte processes and focal/diffuse acute tubular injury. It should be noted that three of the six patients had HRG-APOL1, and none of them had evidence of viral particles on the biopsy. Thus, validating the hypothesis of the “two hits” combination mechanism–genetic predisposition and cytokine-mediated host response to COVID-19, as an important etiological factor (12).

A multicenter study evaluated 284 renal biopsies (240 native kidneys and 44 grafts) from patients infected with COVID-19 (240 native kidneys and 44 grafts) from March 2020 to March 2021 in the USA, India, and Switzerland. Statistical analyses showed that COVAN was the most prevalent finding, present in 62 (25.8%) patients, among which 91.7% were associated with HRG-APOL1 (18).

Table 1 show native kidney biopsy findings in adult and pediatric patients with COVID-19 from a literature review of case series reports. This review involved multicentric and unicentric studies from the USA, India, Switzerland, Italy, and France. Case reports and small series (< 5 cases) were not included.

**TABLE 1:** Biopsy findings of native kidney in patients with COVID-19: Review of case-series reports (*n* = 331) (14, 18, 62–67).

Diagnosis	Number of cases	%
Collapsing glomerulopathy	94	28.4
Acute tubular injury	46	13.9
Diabetic nephropathy	32	9.7
Focal segmental glomerulosclerosis (FSGS)	25	7.6
Minimal change disease	18	5.4
Membranous nephropathy	15	4.5
Pauci-immune crescentic glomerulonephritis	13	3.9
Thrombotic microangiopathy	12	3.6
Infection-associated glomerulonephritis (GN)	9	2.7
Myoglobin cast nephropathy	9	2.7
IgA nephropathy	9	2.7
Arteritis/Arterionephrosclerosis	9	2.7
Lupus nephritis	7	2.1
Amyloidosis	5	1.5
Proliferative glomerulonephritis with monoclonal IgG deposits	4	1.2
Acute interstitial nephritis	4	1.2
Cryoglobulinemic glomerulonephritis	3	0.9
HSP nephritis	2	0.6
Cortical infarct	2	0.6
Anti-glomerular basement membrane antibody disease	2	0.6
Acute pyelonephritis	2	0.6
Light chain cast nephropathy	2	0.6
C3 glomerulonephritis	1	0.3
Membranous-like glomerulopathy with monoclonal IgG kappa deposits	1	0.3
Fibrillary glomerulopathy	1	0.3
Light chain deposition disease	1	0.3
Hemoglobin cast nephropathy	1	0.3
Thin glomerular basement membrane disease	1	0.3
Sickle cell nephropathy	1	0.3

COVID-19, coronavirus disease 2019; HSP, Henoch-Schönlein purpura.

### Membranous nephropathy

Few cases of membranous nephropathy (MN) in the context of the COVID-19 pandemic have been described to date. In a multicenter study carried out in three countries, only 11 (4.6%) of 240 native kidney biopsies showed MN, four of them with positive phospholipase A2 receptor (PLA2R) (18). Kudose et al. detected MN in five (6.6%) of 76 infected patients, and PLA2R was positive in two of them (17). In one reported case, a patient presented MN PLA2R positive 4 weeks after diagnosis of mild COVID-19, with nephrotic syndrome, AKI and partial response to immunosuppressive treatment (19).

However, PLA2R was negative in most MN cases associated with COVID-19, strengthening the hypothesis that glomerular involvement is secondary to the infectious condition (10, 17, 18). The pathophysiological mechanisms are still unclear; however, MN in the context of SARS-CoV-2 infection may be secondary to an exacerbated immune response against the virus (10). Most experts propose postponing immunosuppression in cases of MN without changes in renal function or other complications; nevertheless, there is still little data on the clinical course and outcomes related to MN in the context of COVID-19 (19).

### Immunoglobulin A nephropathy

IgAN is the most common glomerular disease worldwide (20). Some cases of COVID-19-associated IgAN have been reported in the literature since the pandemic began. Huang et al. reported a case of a 65-year-old woman with dark urine, renal dysfunction, and proteinuria after COVID-19 infection, with biopsy-proven IgAN and complete recovery after 3 days of glucocorticoids and angiotensin II receptor blockers therapy (21). Another study reported the case of a patient with AKI, nephrotic proteinuria and hematuria 3 weeks after COVID-19 infection. Renal biopsy was compatible with acute IgA-dominant infection-associated glomerulonephritis, and virus was detected in renal tissue with immunohistochemistry assay (22). In two large series evaluating kidney biopsies from patients with COVID-19, the frequency of IgAN was similar, being diagnosed in 2.6 and 2.9% of cases (17, 18). Apparently, the inflammatory environment and the “cytokine storm” provided by SARS-CoV-2 infection work as triggers in predisposed individuals (22).

### Minimal change disease

In a multicenter study by May et al. only 11 patients were diagnosed with minimal change disease (MCD), corresponding to 4.6% of the evaluated cases (18). Yamada et al. (23) reported a case of MCD-like podocytopathy in a 49-year-old patient who had undergone a kidney transplant 25 years earlier. The patient developed nephrotic syndrome and worsened renal function after the COVID-19 infection. Intense effacement of podocyte processes with microvillous transformation was revealed by electron microscopy. After treatment with glucocorticoid and an angiotensin II receptor inhibitor, clinical improvement was observed, but with persistently elevated proteinuria for up to 6 weeks (23). Although the exact mechanism of MCD is not known, it is possible that the pathogenesis is related to T-lymphocyte activation and cytokine release triggered by viral infection. In a study that evaluated glomerulopathies during COVID-19, MCD was present in one case of 17 patients (14); this was the first case described in the literature of MCD associated with HRG-*APOL-1* in a COVID-19 patient and revealed the presence of “interferon footprints,” demonstrating the importance of the role of cytokine-mediated podocyte injury in predisposed individuals.

### Lupus nephritis

Previous studies have demonstrated a strong association between viral infections with mimicry, such as Epstein–Barr virus, cytomegalovirus, parvovirus B19, and HIV, and the emergence or reactivation of systemic lupus erythematosus (SLE) (24). The mechanism of SARS-CoV-2 infection inducing lupus nephritis may be related to the triggering of an intense immune response with the massive release of inflammatory cytokines such as interferon-gamma, tumor necrosis factor-alpha, interleukin-2 (IL-2), IL-6, IL-7, and IL-10, associated with the production of autoantibodies such as anti-cyclic citrullinated peptide antibody and antinuclear factor antibodies (25).

In the case series reported by Kudose et al. one case (7.14%) case of lupus nephritis (class IV + V) was detected (17). In contrast, May et al. detected six cases of lupus nephritis, corresponding to 2.5% of the native renal biopsy results (18); among these, three, two, and one were characterized as sclerotic lupus nephritis, membranous lupus nephritis, and minimal mesangial lupus nephritis (lupus podocytopathy), respectively. In a study published by Zamani et al. a patient diagnosed with SLE and lupus nephritis class I after COVID-19 underwent pulse therapy with glucocorticoids for 3 days plus monthly infusions of cyclophosphamide and daily oral prednisone (25). The patient was discharged with improvement in symptoms, proteinuria, and normalization of anti-DNA levels after 6 months.

### Pauci-immune crescentic glomerulonephritis

Crescentic glomerulonephritis was not described among the 14 biopsies from patients with COVID-19 reported in a study published by Kudose et al. (17). However, in a multicentric publication by May et al. (18) 11 crescentic glomerulonephritis cases were found, eight of which were positive for antineutrophilic cytoplasmic antibody (ANCA) (18). Crescentic glomerulonephritis results from a wide range of disease associated with immune dysregulation (26). In a study published by Uppal et al. (27), two patients were diagnosed with pauci-immune crescentic glomerulonephritis (PICGN) a few days to 2 weeks after COVID-19 infection. Both patients received a glucocorticoid pulse followed by an infusion of rituximab after a negative reverse transcription-PCR for SARS-CoV-2 and showed improved renal function and symptoms after the first month of rituximab doses (27).

One of the mechanisms proposed to explain the emergence of this subtype of glomerular injury would be related to the uremic state associated “cytokine storm,” which could lead to an inadequate response in the face of an infectious condition, culminating in an ANCA-associated vasculitis (26, 27). Another related mechanism would be that the host’s factors predispose to certain types of renal pathologies due to a “second hit” (assuming SARS-CoV-2 infection) (27).

### Injuries of tubulo-interstitial compartment

Acute tubular injury was the principal diagnosis in six of 17 patients with COVID-19 who underwent biopsy in a series of cases including native kidneys and allografts. Four patients had exposure to potentially nephrotoxic drugs and one patient had rhabdomyolysis with pigmented cast (14). In these cases, the etiology of kidney injury is multifactorial and complex, including sepsis, hypoxia, hemodynamic instability, nephrotoxin exposure, and multiorgan complications (14).

The kidney tissue is rich in ACE2 receptors and is characterized as one of the main targets of infection by the new coronavirus. The main site of these receptors in the kidneys is the apical membrane brush border of the proximal tubules and, to a lesser extent, in podocytes (9).

Direct cytotoxic action of the virus in the tubules has already been described as the main etiology among the probable mechanisms of tubular injury, resulting in mitochondrial dysfunction, acute tubular necrosis, tubular proteinuria, and hematuria (14). Another mechanism presented as a cause of tubular dysfunction in COVID-19 infection is acute interstitial nephritis, which, despite not being a rare cause of AKI, remains poorly recognized and diagnosed in the context of viralus infection. The main proposed mechanisms are be the direct action of the virus on the tubules and indirect action secondary to medications or other factors associated with the virus (5). In contrast, despite the most recent findings, viral particles were not directly detected in the renal tissue in a large multicenter study of 284 patients who underwent renal biopsy, supporting the hypothesis that the lesion has a multifactorial etiology (18).

Rhabdomyolysis has also been described as a possible cause of AKI in patients infected with COVID-19, associated with the need for RRT and high mortality (28, 29). Despite the multifactorial etiology, systemic muscle damage caused by the direct action of the virus, “cytokine storm,” and the hypoxemia environment seem to be the main causes (18, 28–30). Muscle injury triggers the release of large amounts of myoglobin-containing heme pigment, which can obstruct the tubular lumen, culminating in acute tubular necrosis (30).

Although the main hypothesis for the etiology of tubular damage is ischemia secondary to shock, e studies have demonstrated the presence of acute tubular necrosis in the absence of hemodynamic compromise or severe pulmonary alterations. Therefore, the hypothesis that the tubular lesion is predominantly ischemic does not seem to cover all cases with acute tubular necrosis, presenting the possibility of direct viral cytotoxic action as the main mechanism in some cases (31).

### Vascular injury

Numerous cases have shown that the infection with the new coronavirus can lead to a prothrombotic inflammatory state, culminating in arterial or venous thrombosis with diverse clinical manifestations and outcomes (32, 33). Reports of stroke, AKI, and systemic and coronary vasculitis in COVID-19 have increased (34). The coagulopathy scenario generated by COVID-19 is usually evidenced by changes in tests, such as prothrombin time and D-dimer and fibrinogen serum levels.

The mechanism associated with a prothrombotic inflammatory state is possibly related to endothelial injury secondary to the activation of macrophages and monocytes and the release of inflammatory mediators, culminating in platelet activation, thrombin generation, and fibrin clot formation (9, 33). Another proposed mechanism to justify the procoagulant state is the activation of the complement system, causing significant damage to the microvasculature. There are also reports of strong evidence of systemic thrombophilia and microvascular injury associated with elevated plasma levels of C5d and endothelial deposits of C5b-9 in patients with COVID-19 (34).

The presence of endothelial dysfunction, activation of the coagulation cascade, and microcirculation thrombosis in the kidneys may be risk factors for AKI (9). The prevalence of thrombotic alterations in renal tissue was also described in a multicenter study, in which five cases of thrombotic microangiopathy were described among 240 native kidney biopsies analyzed (18). There is a correlation between COVID-19 infection and systemic vasculitis with different patterns; however, the mechanisms have not yet been well established. Thrombosis cases in the pulmonary vascular vessels, distal to the alveolar capillary bed, which work with a “clot filter,” may not be secondary to the systemic microembolism scenario but to a similar scenario as the vasculitis related to COVID-19, with repercussions on various organs, including the kidneys (32–34).

In a series of seven autopsies of patients who died from COVID-19 found cases of fibrin-rich microthrombi in scattered peritubular capillaries, thrombotic microangiopathy with large platelet-rich microthrombi, and microhemorrhage in the interstitium, as well as virions in proximal convoluted tubules and podocytes (35).

### Comorbidities and severity of SARS-CoV-2 infection at the time of kidney biopsy

Most patients (70%) were symptomatic and had a moderate-to-severe disease at the time of kidney biopsy, and comorbid diseases were very common (85%). Therefore, these conditions can be considered risk factors for developing kidney injuries described above. Moderate-to-severe infections refer to infections requiring hospitalization, supplemental oxygen, intensive care, mechanical ventilation, and dialysis. The most frequent comorbidities were hypertension, diabetes mellitus, and obesity.

## Morphological findings in renal involvement post COVID-19 vaccine

More than 8.2 billion COVID-19 vaccine doses have been administered globally to contain the contamination and mortality curve of the new coronavirus, resulting in a substantial reduction in the number of cases and deaths in several countries (36). Although adverse renal effects are rare in the context of COVID-19 vaccination, some cases of adverse effects in various organs, including the kidneys, have been reported and have been of concern to nephrologists (37).

It is well established that the immune response generated by the most diverse vaccines, such as vaccines against meningococcus C and B virus, influenza, and diphtheria-tetanus-pertussis (DTP), is a potential trigger for developing or reactivating nephrotic syndrome (36–40). However, in the most recently published studies related to post-vaccination conditions against COVID-19, in addition to nephrotic conditions, other forms of renal involvement have been reported, such as acute tubular necrosis and thrombotic microangiopathies (Table 1). In this sense, the nephrological community and other specializations remain vigilant regarding the evolution of cases of patients with post-vaccine involvement to better understand the mechanisms and associated outcomes (7, 39).

### Glomerular injury

Although all vaccines against the new coronavirus are related to glomerular conditions, most studies have indicated that vaccines based on messenger ribonucleic acid (RNA) (Pfizer-BioNTech BNT162b2 and Moderna mRNA1273) are the most prevalent (38). The characteristics of post-vaccination COVID-19 kidney injury, based on the literature review, are summarized in Table 2.

**TABLE 2:** Characteristics of post-vaccination COVID-19 kidney injury based on literature review (*n* = 82).

Glomerular injury	*N*	Vaccine	Number of cases per dose	Time from vaccine to diagnosis
IgA nephropathy (7, 39, 40, 68–72)	27	Pfizer–12	1st dose: 5	3 h to 6 weeks
		Moderna–10	2nd dose: 22	
		Sinopharm–1		
		Astrazeneca–1		
		RNAm*–3		
Minimal change disease (6, 7, 39, 40, 44, 54, 55, 63, 73–75)	21	Pfizer–11	1st dose: 11	2 days to 4 weeks
		Moderna–7	2nd dose: 8	
		Astrazeneca–2		
		RNAm*–1		
Crescentic glomerulonephritis (40, 42, 76–79)	12	Pfizer–6	1st dose: 2	2 days to 4 weeks
		Moderna–5	2nd dose: 8	
		Covaxin–1		
Membranous nephropathy (7, 40, 80)	7	Pfizer–3	1st dose: 2	1 day to 4 weeks
		Moderna–3	2nd dose: 5	
		Johnson–1		
		Moderna–5		
Lupus nephritis (40, 80–83)	5	Pfizer–2	1st dose: 4	2 days to 1 week
		Astrazeneca–2	2nd dose: 1	
		Moderna–1		
Collapsing glomerulopathy (37, 38)	4	Moderna–2	1st dose: 2	1 s to 3 weeks
		Astrazeneca–2	2nd dose: 2	
Anti-glomerular basement membrane (7, 39)	3	Pfizer–2	1st dose: 1	1 day to 2 weeks
		Moderna–1	2nd dose: 2	
Focal segmental glomerulosclerosis (7)	1	Pfizer–1	2nd dose: 2	3 weeks
Scleroderma renal crisis (38)	1	Pfizer–1	1st dose: 1	1 day
C3 glomerulonephritis (84)	1	Astrazeneca–1	1st dose: 1	1 week

*Pfizer and Moderna were the available vaccines at the time of publication.

In a series of published cases with 13 patients who developed kidney damage after using a messenger-RNA-based vaccine, eight (62%) patients presented with newly diagnosed glomerulopathy, and five (38%) presented with reactivation of previous conditions. In this study, the most common glomerulopathy was IgAN (38%), followed by MN (23%) and podocytopathies (23%) (7). In another case series, patients who received messenger RNA-related (27) and adenovirus (2) vaccines showed changes in renal function and glomerular syndrome after 1 month. The main findings in the biopsies were IgAN (10), MCD (7), CG (2), crescentic glomerulopathy (6), MN (3), and lupus nephritis (1) (38). The most frequent presentation of glomerular injury post-COVID-19 vaccine was AKI with nephrotic or nephritic syndrome and gross hematuria, followed by nephritic syndrome and nephrotic syndrome with preserved renal function (7, 38).

The onset of glomerulonephritis usually occurs after 3 weeks of immunization, most of which occur within the first week (37). The activation time of IgAN was 1–2 days after receiving the 2nd dose of Pfizer-BioNTech BNT162b2 and Moderna mRNA1273, while MCD usually appeared on the 7th day after the 1st dose, suggesting a direct effect of immunization on the emergence of these two diseases (38). Furthermore, the work published by D’Agati et al. corroborates the hypothesis of the direct action of vaccines on the glomerulus since there is a strong temporal association between receipt of the vaccine and the onset of symptoms, suggesting a rapid response mediated by T-cells as a trigger for podocyte lesions (41).

Most patients with post-vaccine glomerulopathy were treated with immunosuppression according to histopathological diagnosis. In one case series, twenty-seven patients were treated with immunosuppression and followed up. Eight of them recovered renal function completely, five showed partial recovery, and fourteen did not improve, and five of them required hemodialysis (38). In another study, ten of thirteen patients were followed, nine of whom received immunosuppression. Eight patients responded to treatment (six with immunosuppression and two with conservative treatment) (7).

### Vasculitis

Vaccine involvement in ANCA-related vasculitis may be systemic or limited to the renal system (39). In a review of 29 cases of ANCA-related vasculitis after COVID-19 vaccination, 24 cases were diagnosed after the vaccine, and the remaining cases had a recurrence or worsening of pre-existing vasculitis (40). Cases occurred 2–37 days after immunization, with the majority being associated with the RNA vaccine. The most frequent antibody was myeloperoxidase-ANCA (15), but proteinase 3-ANCA (3), double positivity (3), and anti-glomerular basement membrane were also found (40); approximately 50% occurred after the first dose and 50% after the second dose, and it could even occur after both doses (39, 40).

Kidney injury was present in 93% of the vaccine-associated vasculitis cases. The most common histopathologic findings were crescentic glomerulonephritis and fibrinoid necrosis without endocapillary proliferation and deposits on immunofluorescence (40). There was also a reported case of myeloperoxidase-vasculitis and rhabdomyolysis after Pfizer-BioNTech COVID-19 mRNA vaccination, whose biopsy demonstrated PICGN in addition to a severe acute tubular lesion with myoglobin cast and interstitial inflammation (42).

The exact mechanism of COVID vaccine-associated vasculitis is not fully understood. In addition to molecular mimicry mechanisms, RNA vaccines can lead to aberrant activation of the innate and acquired immune systems that, especially in genetically predisposed individuals, can serve as a basis for triggering autoimmune diseases (41–43). Regarding the inactivated vaccines, there may also be an induction of autoimmunity associated with the immune response to SARS-CoV-2 proteins (44). Most cases of COVID-19 vaccine-associated vasculitis tended to respond to immunosuppression as per the usual vasculitis treatment (42–45).

### Rhabdomyolysis

Myalgia is one of the most common side effects associated with different types of COVID-19 vaccines and is often mild and self-limiting (46). More severe cases requiring hospitalization, including myositis and rhabdomyolysis, were less frequent (47, 48). This observation may lead to the belief that reported cases of vaccine-related rhabdomyolysis are underestimated.

A few cases of rhabdomyolysis have been reported, most of which are associated with mRNA vaccines (47–49). However, other vaccines, such as ChAdOx1nCoV-19 (AstraZeneca) and Ad26.COV2.S (Johnson & Johnson) have also been associated with this complication (50, 51). The clinical presentation can vary from mild manifestations without renal dysfunction (48) to severe manifestations with AKI, RRT, compartment syndrome, and death (49, 52, 53). The time between vaccination and symptom onset can vary from 1 to 14 days (50, 51, 53, 54).

Rhabdomyolysis secondary to vaccination has been reported previously, mainly following influenza vaccination (55). The mechanisms associated with this type of adverse effect of the COVID vaccine are not well understood, with some reported cases having potentially confounding factors such as statin use and previous neuromuscular disease (47, 53). However, this may arise from an exaggerated immune response to adjuvants, possibly potentiated by prior exposure to the COVID-19 virus (46, 54). Clinicians should be aware of the possibility of rhabdomyolysis as a complication of COVID-19 vaccination because, in such cases, early diagnosis and intervention, including vigorous hydration and elimination of factors that potentiate AKI, may be crucial for a better prognosis (56).

#### Vaccination recommendations in patients with immune-mediated renal diseases

Despite the adverse effects related to vaccination against the new coronavirus, immunization remains the main tool to control the number of new cases and mortality. Patients with glomerulopathies and proteinuria may be at a higher risk of severe infections, mainly because of the loss of immunoglobulins in the urine; it is important to use available prevention measures (57). However, more studies specific to immune-mediated kidney disease populations are needed.

Consideration should be given to patients’ current disease status and the use of immunosuppression. It is known that patients on immunosuppressants, such as B cell–depleting agents, mycophenolate mofetil, and glucocorticoids, may have reduced humoral response to the vaccine (57–60). For example, if rituximab is used, it may be necessary to delay vaccination for up to 6 months after stopping this medication to allow B-cell reconstitution and maximize vaccine response (59, 60).

Despite the greater number of activated or reactivated glomerular diseases being related to vaccines based on messenger RNA, conclusive data are still lacking for the non-recommendation of these vaccines to the detriment of others (38). For the pediatric population, an mRNA vaccine is recommended considering age restrictions for adenovirus-vectored vaccines and immunosuppression (61). It is also recommended that signs of relapse be monitored after vaccination and that treatment should follow the usual recommendations for the underlying disease (61).

However, available recommendations and data on the relationship between COVID-19 vaccines and kidney lesions are still scarce. Since this is a new disease whose vaccines were developed and applied only in late 2020, the current information is based on case reports and case series. Although case reports are useful for pharmacovigilance and are the first source of evidence for detecting adverse events related to drugs and vaccines, this type of scientific information alone is insufficient to establish a definitive causal relationship between the vaccine and kidney lesions. When analyzing these cases according to Bradford Hill’s causality criteria, temporality, coherence, plausibility, and analogy can be observed. Consistency can also be considered due to repeated events observed in different locations and circumstances. However, not all criteria have been met to date and cannot be used to establish causal relationship.

## Conclusion

Renal involvement caused by COVID-19 has a strong impact on the evolutionary course of the disease, resulting in higher morbidity and mortality rates. This study aimed to elucidate the main forms of renal involvement in the context of SARS-CoV-2 infection, as well as the morphological findings and probable pathophysiological mechanisms involved. The main renal changes were listed in patients who received doses of the most diverse vaccinations against COVID-19. However, despite the aforementioned findings, mass vaccination has proven to be safe in the most diverse studies, constituting the main means of controlling new cases and reducing hospitalization and deaths, especially in the population with chronic kidney diseases.

## Author contributions

IP, NF, GS, and PN: conceptualization. IP, DC, and GS: methodology. IP, GS, and PN: data curation and writing–original draft preparation. IP, DC, DS, NF, GS, and PN: writing–review and editing. DC, GS, and PN: supervision. All authors contributed to the article and approved the submitted version.
